# Insights into abusive workplace behavior

**DOI:** 10.3389/fpsyg.2023.990501

**Published:** 2023-07-28

**Authors:** Aharon Tziner, Hadara Bar-Mor, Daphna Shwartz-Asher, Or Shkoler, Lilach Geva, Haim Levi

**Affiliations:** ^1^Peres Academic Center, Rehovot, Tel Aviv, Israel; ^2^Netanya Academic College, Netanya, Israel; ^3^Tel-Hai College, Qiryat Shemona, Israel; ^4^HEC Montréal, Université de Montréal, Montreal, QC, Canada

**Keywords:** abusive behavior, bullying, abusive behavior outcomes, abusive behavior antecedents, Tepper’s inventory

## Abstract

**Objectives:**

This paper explores definitions, incidences, and outcomes of workplace abuse – a widespread, worldwide social phenomenon impinging on the well-being of workers in a developing labor market – and possible directions for delineating and standardizing classifications of the phenomenon that facilitate workers’ protection. Furthermore, we employ Tepper’s Abusive Supervision Survey Questionnaire [ASSQ] to identify managerial abuse in organizations regarding the type of organization (public/private), gender of the perpetrator, gender of the target, and the managerial role, of each of the abuse items. Finally, we suggest directions for further research and practical measures designed to facilitate the diminution of behavioral abuse in the workplace in the foreseeable future.

**Hypotheses:**

No hypotheses are submitted in this exploratory paper.

**Methods:**

Employees of two large organizations (the Katzrin Local Council and Tigbur Ltd.) and another small national organization in Israel were approached, requesting their assistance in this investigation. We introduced the questionnaire, noting its anonymity and the academic context, and that subject participation was voluntary. The questionnaire consisted of fifteen statements on a five-point response scale ranging from 1 = “I cannot remember him/her ever using this behavior with me” to 5 = “He/she uses this behavior very often with me” (alpha = 0.97, *M* = 1.81, SD = 1.03). One hundred five respondents comprised the final sample: men (39%) and women (61%) aged 23–66. 66.7% of the respondents were employed in a public organization, 28.5% in a private organization, and 4.8% in a national organization. Years of education: less than 12 (14.3%); 12 full years (17.1%); tertiary education (10.5%); bachelor’s degree (40%); and master’s degree and above (18.1%). While 28.6% held managerial positions, 71.4% did not. Job tenure ranged between 0.5 and 42 years. The participants in national and private organizations were combined to avoid the problem of unbalanced groups. Notably, the gender balance of the managers was men (50.5%) and women (49.5%). To ensure statistical validity, we conducted a factor analysis and performed Pearson correlations to examine the relationships between the general latent variable and the abuse items and their intercorrelations. Additionally, we conducted *t*-tests for independent samples (with Bonferroni corrections for multiple comparisons: see, for example) to compare (1) the respondent’s gender; (2) manager’s gender; and (3) the managerial role with each of the abuse items, and (4) the type of organization (private/public); including the general abuse variable.

**Results:**

High, positive, and significant correlations were recorded between each questionnaire item and the total score of abusive behavior. The results ranged from *r* = 0.92 for the statement, “Puts me down in front of others” (*r* = 0.92) to *r* = 0.69 for the statement, “Does not allow me to interact with my co-workers” (*r* = 0.69). Reported abuse in the respondents’ organizations was relatively low (1.81), while among women managers marginally less than their male equivalents (in absolute value, the mean difference in the mean scores fell between 0.28 and 1.25). Abusive behavior in private organizations was more prevalent than in public concerns. Not surprisingly, workers reported more incidents of abuse than managers: in absolute value, the mean difference between employees and managers fell between 0 and 0.67 in the mean scores. Women reported fewer abusive behavior incidents than men; however, the differences found were insignificant.

**Conclusion:**

The reports of abusive behaviors were meager. Explanations include: (1) Any instance of alleged workplace abuse can be interpreted variously by different individuals, a function of subjective perceptions and interpretations of objective data informed by several internal and external factors impinging on workers’ wellness at work. (2) The extant “trust gap,” which is part of the pervading culture, mitigates against honest reporting of workplace abuse for fear of reprisals. (3) A single report of behavioral abuse at work may be attributable to the same perpetrator repeating the aggression several times. The lower incidence of reported abuse among female managers could be understood in line with studies that indicate that men display aggression more frequently than women playing out their expected respective stereotypical roles in society. Furthermore, following women reflect more stable personality dispositions and are less likely than men to aggress against others without provocation. In line with these observations, women’s management style projects “an ethics of care,” focusing on interpersonal relations and a greater tendency than male managers toward cooperation, creativity, innovation, and shared decision-making. Because the differences found in all the variables in our investigation investigated were not significant, we cannot conclude that the results indicate a trend. Finally, we assume that appropriate sanctions against perpetrators at the workplace and regulation in public organizations would reduce the incidence of workplace abuse. In a more optimistic vein, we recommend that management and HR personnel initiate positive measures to raise awareness of behavioral abuse and eliminate it from their organizations. They should initiate training workshops, events, and support groups akin to those extant in organizations focused on eliminating racial prejudice and advocating sustainability and wellness in the workplace. Ultimately, the goal is to protect workers’ dignity, the integrity of the organizations, and the welfare of society.

## 1. Introduction

Work is an inseparable part of human lives. Through work, human beings fulfill a large part of their needs and inspirations. Therefore, the atmosphere and relationships at work are extremely important. A positive and healthy atmosphere in the workplace is necessary for the well-being of employees while the impact of a workplace disruption (e.g., abusive supervision) on working patterns has a negative influence on all work actors. This antagonistic behavior is not only unacceptable in the psychological domain, but is also forbidden by the law (see, for example, Hoel et al., 2000; Tepper, 2000; Cortina and Magley, 2003; Tepper et al., 2008).

Undoubtedly, workplace abuse is a growing phenomenon, a prime reason for the importance of scrutinizing it. For example, the International Labour Organization (ILO, 2020) reported that 26% of workers worldwide have experienced workplace violence or harassment in the past year. This statistic is up from 22% in 2010. ILO’s study also found that women are more likely to experience workplace violence or harassment than men. Other examples include (A) a study by the Workplace Bullying Institute (2018) that found that 60% of American workers have experienced workplace bullying; (B) a study by the European Union Agency for Safety and Health at Work (2019) that found that 55% of European workers have experienced workplace harassment; and (C) a study by the Australian Human Rights Commission (2020) that found that 40% of Australian workers have experienced workplace bullying.

Generally, despite the crystal-clear association between them, these two fields rarely intertwine academically. Reasons stem from different research goals and foci, different research methodologies, funding issues, and more. A call for more interdisciplinary research on workplace abuse was given as early as 2005, and to this date, and to the best of our knowledge, none has picked up the gauntlet of this crucial workplace phenomenon. That is to say, labor law and occupational psychology scholars can learn from each other and may help develop more effective interventions to prevent and address workplace abuse (Fitzgerald and Shullman, 1993).

Specifically, however, by studying the phenomenon of abusive workplaces, researchers may gain better and finer insights into its prevalence, causes, consequences, potential interventions, legal implications, obligations, and protections surrounding workplace abuse, in addition to the psychological processes, individual and organizational factors, and the impact of such behaviors on employees and organizations. It is the point where both occupational psychology and labor law domains collide and intertwine. The importance of such interdisciplinary endeavors cannot be overstated, as there is a plethora of vital implications to exploring this phenomenon further, for example (non-exhaustive list): (1) organizational productivity and performance; (2) employee performance and productivity; (3) legal and ethical considerations; (4) cost implications; (5) social responsibility; (6) employee protection; (7) promoting fair and inclusive work environments; (8) assessing legal remedies; (9) employer liability and risk management; (10) psychological well-being of employees; (11) work engagement and motivation; (12) organizational climate and culture; (13) leadership and management practices; and more (e.g., Bowling and Beehr, 2006; Nielsen and Einarsen, 2018a). This phenomenon is of paramount importance to a healthy society and economy.

Abusive behavior (e.g., bullying) in the workplace is a very dangerous issue both to the employer and the employees and can be traced and found in every organization. The main component of this phenomenon is its identification as abuse since different types of daily behaviors cause stress and mental suffering. Workplace abusive behavior can take different forms., for instance, bullying.

Bullying among co-workers (not only of superiors to their subordinates) includes physical abuse, intentionally spreading false information to hurt colleagues at work; telling inappropriate jokes in the workplace that can offend others; telling false stories about colleagues to humiliate and intimidate them. Bullying against inferior employees under supervision includes physical abuse or threats of such abuse; consistently insulting and criticizing employees; belittling and devaluing the opinions of others in a collaborative or discussion setting; excluding or ignoring an employee in the office or even at social events; acting aggressively either physically or by abusing the intimidator’s position; intentionally assigning too challenging or even impossible tasks to complete to justify punishments; spying, or invading the privacy of inferiors by hacking into their emails (Indeed Editorial Team, 2023).

Workplace bullying (WPB) has been considered an important social problem for over four decades (Salin and Notelaers, 2017). It is costly to organizations and individuals and is considered one of the most difficult areas for human resource (HR) professionals to manage (Catley et al., 2017).

Workplace abuse represents one form of workplace mistreatment behaviors such as violence, aggression, bullying, and incivility (Nielsen and Einarsen, 2018b). Tepper et al. (2017) estimated that approximately 10% of employees experienced supervisory abuse. Bowling et al. (2015) reported that 10 to 41% of employees faced workplace aggression acts in the United States, as well as 8 to 26% in Austria, 3 to 20% in Belgium, 2 to 27% in Denmark, 5 to 24% in Finland, 8 to 10% in France, 23% in Ireland, 5 to 9% in Lithuania, 20% in South Africa, 4% in Sweden, 55% in Turkey, and 11% in the United Kingdom. Therefore, workplace aggression is a global issue (Caillier, 2021). Feldblum and Lipnic (2016) claimed that as many as 50% of U.S. women experience sexual harassment during their working lives, but only a minority report it.

Anyone connected to the organization’s employees can be the aggressor, including managers, coworkers, subordinates, and even nonorganizational members, such as customers, clients, patients, and contractors. Consequently, workplace aggression is common (Caillier, 2021). Tepper et al. (2017) suggested that supervisors were the leading sources of abuse in organizations.

Ramdeo and Singh (2019) investigate the goal of understanding abuse from different sources by comparing the consequences of abusive supervisor and abusive co-worker behaviors. They explain the process by which procedural justice perceptions shape employee reactions to workplace abuse. Their study intends to demonstrate the relevance of the social exchange theory to the domain of abusive co-workers and further expand our understanding of procedural justice perceptions. De Cieri et al. (2019) study examined the impact of power imbalance in organizational relationships and claim it explains associations between perpetrators and those who experience bullying.

Stress, anxiety, and depression, as well as lower levels of work attitudes (Manier et al., 2017), are a few of the adverse employee outcomes that come from workplace aggression. Workplace abuse and stress are related to poorer mental health, including sleep disorders, depression, anxiety, post-traumatic stress disorder and symptoms, and psychological distress (Nabe-Nielsen et al., 2016). This can be the case even for co-workers who are not directly victimized (Di Marco et al., 2016, 2018). Exposure to workplace stress has also been associated with increased musculoskeletal injuries and disorders and a higher cardiovascular risk score among flight logistic workers and flight attendants (Lecca et al., 2018).

The systems that are currently in place have proven insufficient to prevent workplace abuse (Burke and Cooper, 2018), and its victims are often left without support, within their job or from clinicians, while navigating the fallout of these experiences (Gale et al., 2019).

Recent studies (e.g., Liao et al., 2021) indicate that abusive workplace behavior is a widespread social phenomenon that cannot be ignored or trivialized. The issue is prevalent, for example, in countries ranging from the U.S. and Canada to Europe, the UK, Israel, and South Africa. The importance of addressing this issue is evident, given the link between the quality of employment and mental and physical health, and developments in the labor market (Dumitru, 2016). The latter include globalization processes, the COVID-19 epidemic’s influence on economic development, technological innovation, and changes in labor market demographics (Radulescu et al., 2020; Li et al., 2022).

Existing theoretical frameworks to define abuse are based on two approaches: (1) harm to a person’s dignity and (2) harm to the physical and mental health and safety of the individual (Hodson, 2001; Keashly, 2001). However, these two aspects insufficiently capture the injustice involved in workplace abuse; the framework does not sufficiently provide guidelines to those who counter workplace abuse, whether management, policymakers, and regulators. Indeed, these areas of research and inquiry are relatively new in the research field (Escartín, 2016).

In general, however, it can be said that abusive behavior in the workplace is inappropriate conduct that causes damage to people’s dignity, and mental and physical health and safety (see Hawkins v. United States, 2019). Following Guerrero (2004), there is, consequently, a striking need to set appropriate behavior norms in the workplace. Furthermore, we would add that all parties associated with eliminating workplace abuse cooperate in standardizing its definition to ease these guidelines into court legislation, where necessary. Notably, protecting human dignity and freedom is one of the essential constitutional values of most legal systems; accordingly, the law is committed to preventing such harm.

Abusive workplace behavior touches upon individuals, but other units in the workforce are likewise exposed to its nefarious consequences. Subsequently, although many concerns prefer to keep these issues among themselves, we propose that because of the severe damage to people’s lives and livelihood, the treatment of the phenomenon cannot remain solely at the organizational level.

Abusive behavior in organizations appears to be a widespread phenomenon. Although previous publications attempted to shed light on the causes and mechanisms through which it develops globally (Harvey et al., 2007, 2009), they (1) have not addressed the full range of abusive behaviors but rather solely bullying, and (2) have not looked into particular difference factors (e.g., gender, type of economic sector such as private vs. public organizations). In this paper, we present a brief overview of several aspects of workplace abuse in the hope that the paper will enhance awareness of the issue and the desired outcome of increasing workers’ protection and keeping their dignity. Moreover, we conducted an empirical investigation to examine the association between two difference factors (gender and private/public organizations) and the manifestations of abusive behavior of superiors to subordinates, using Tepper’s inventory. The underlying thinking was that if the instrument proves effective in detecting distinct associations between the gamut of abusive behaviors and different organizational factors, it could be used as a diagnostic tool to prevent the phenomenon of abusive behavior.

### 1.1. Components of abusive workplace behavior

Work relationships can sometimes be painful and lead to actions that are inappropriate at least and, at most, abusive and violent. Nobody is excluded from this phenomenon, whether they are employers, managers, supervisors, or employees. The latter includes regular employees and other workers, irrespective of contractual status. Indeed, even trainees, whether interns or apprentices and contingent workers such as independent contractors and freelancers are subject to abuse in their respective places of work.

Although there is no universally accepted definition of workplace abuse (Schneebaum, 2021), an observer can detect the hostility expressed in many ways. These damaging activities include psychological abuse; humiliation; ostracizing fellow workers; sabotaging colleagues’ work; slandering colleagues to co-workers or superiors; and shaming co-employees in social media groups.

Examples of workplace abuse abound. They include shouting, overly harsh or unjustified criticism, threats, intimidation, and excessive monitoring. Of note, despite that during the COVID pandemic, work interactions were transformed because so many people worked from home, abuse nevertheless continued and intensified on the electronic networks (e.g., Stephenson et al., 2018; Ferrara et al., 2020). On-screen communications allow people to be less inhibited than during face-to-face dialog. However, offenses on social media can be easily documented and thus provable and should undoubtedly be incorporated into definitions of workplace abuse.

Electronic communication, however, leaves scope for ambiguity and represents but one of several unclear representations or contexts of what constitutes workplace abuse. Consider, for instance, that such abuse incorporates direct and indirect negative behaviors that embody intimidation, hostility, and harm. Generally persistent, the abuse is manifested by individuals or groups toward other persons or groups at work. Moreover, the molestation can occur in a public or private domain, in real-time, or virtually (D’Cruz, 2015; D’Cruz et al., 2018).

Adding to what we have labeled “ambiguity,” we might consider that while abusive behavior at work cuts across all sectors and genders (Heilbrunn and Itzkovitz, 2017), those actions might occur at varying frequencies. To what extent does frequency circumscribe the negative quality of abusive behavior? Similarly, some recorded manifestations of workplace abuse, such as excessive workloads, tend to be more common than others, while others are rarer, such as threats of physical violence. To what extent, we might question, are the two cases equal in severity?

To add to the lack of clarity: workplace abuse is sometimes labeled as harassment or emotional abuse (Fox and Spector, 2005). Following D’Cruz and Noronha (2016, p. 409), known types of emotional workplace abuse incorporate abuse that is interpersonal or depersonalized (pp. 412–413) that, in turn, may be internal or external to the workplace (p. 414). As indicated, this typology is consistent with face-to-face and cyber communication described as traditional and virtual bullying (see also D’Cruz, 2015, p. 8; D’Cruz and Noronha, 2013).

Because of the various representations and manifestations of workplace abuse, we find it helpful to group them into three major categories – incivility, harassment, and bullying.

- Incivility. Following Anderson and Pearson (1999), incivility is low-intensity, interpersonal, deviant behavior. Offenders scorn their targets, doubt their judgment, and address them unprofessionally with rudeness and disrespect. By way of illustration, a survey questionnaire consisted of twelve manifestations of supervisor incivility that included such items as “Shouted at you,” “Ignored or failed to speak to you,” and “Accused you of incompetence” (Cortina et al., 2013). When targets were subjected to these incivilities over an extended period, victims expressed low job satisfaction, increased withdrawal from work, and intention to quit (Cortina et al., 2001, 2013; Mackey et al., 2019).
- Harassment. When abusers systematically and repeatedly manifest unethical behavior that makes the targets feel helpless and unable to counter the victimization, we call this harassment. Harassment is manifested, among other ways, by character defamation, unreasonable criticism, and excessive monitoring of work performance. Lee et al. (2016) observed that harassment affects targets’ mental and physical health, irrespective of race, religion, gender, age, or disability.
- Bullying. Bullies consistently display harmful verbal and nonverbal behaviors over an extended period. Instances include one or more of the following: socially excluding; ignoring the target person; persistently and intentionally offending and insulting; deliberate and frequent emotional abuse; humiliation (privately or in public); gossiping and spreading rumors (Lee and Lim, 2019). Bullying may occur due to several factors, including low self-esteem, stress, or the bully’s sallied life history. Victims of bullying (like victims of harassment) are most often selected (but not exclusively) by their bullies based on race, religion, gender, age, or disability (Ditch and Label, 2021). Among the crushing effects of bullying on targets are high stress and anxiety levels, sleep impediments, depression, and even suicidal thoughts (Lipinski and Crothers, 2013).

Workplace abuse is thus a multifaceted concept. Furthermore, there are international dilemmas and peculiarities involved in its delineation. Notably, research to delineate workplace abuse accurately is in its preliminary stages and has yet to achieve the conceptual analysis that sexual abuse has engendered. We believe, however, that accurate delineation of abuse that informs an employee’s quality of life at work should ultimately be based on three foundations: physio-psychological, administrative-sociological, and legal.

The physio-psychological foundation deals with aspects of personal-mental, physical, and safety-related injury of the kind discussed so far. The administrative-sociological foundation is anchored in Schneebaum’s (2021) proposal that the state should regulate the power embedded in the authority of office rather than being left to the mercy of self-regulation: the employers should not be free to determine whether and to what extent internal authority positions should be regulated. To prevent abuse, the legislator should dictate a web of authority relationships that neutralizes the empowerment of employers and other positions of power (Schneebaum, 2021).

The legal-constitutional aspects of damage to human dignity are the meat of the legal foundation. While the theoretical foundations for examining workplace abuse as legal harm are usually not addressed directly in the literature (Schneebaum, 2021), there are, however, two core frameworks for conceptualizing workplace abuse within that conceptual framework. The first defines workplace bullying as a safety issue dealt with by health and safety regulations focused on preventing injuries and burdening the employer with the responsibility for all risk-creating practices. The second framework focuses on the humiliation of the bullied persons and identifies abuse based on the right to dignity (Schneebaum, 2021).

Although the administrative and legal aspects of workplace abuse go beyond the scope of this paper, we propose, nevertheless, that this trinity of foundations should help to accurately outline the borders of the definition and serve to operate the mechanisms that identify workplace abuse on the local, national, and international arenas.

Finally, in the interests of economy and brevity, we employ the catch-all term “abusive behavior” to describe these negative actions in the workplace.

## 2. Various facets of workplace abuse

### 2.1. The trust gap, antecedents, and outcomes of workplace abuse

#### 2.1.1. The trust gap

Although abusive behavior is commonplace worldwide, employees hesitate to complain about their supervisors because they fear reprisals. This “trust gap” is particularly prevalent in the workplace because expressed grievances are frequently ignored and not treated with the appropriate level of concern and responsibility.

For example, Vault Platform (2021) conducted a study of both American and British office workers. They noted that the trust gap not only applied to workers unwilling to say their piece for the reasons stated above but also that their employers were worried about damage to their reputation and jobs in the light of complaints about abuse. Of note was their comment concerning the gap between workers’ expectations of the workplace and reality at work (Vault Platform, 2021, p. 2) which raises the question of subjective versus objective perceptions of abuse and the degree of offense that constitutes mistreatment. Besides the psychological effects and related concomitants on productivity at work, we might add that the answers to these questions have consequences in courts of law where cases of abuse are heard.

By way of illustration, we might ask to what extent overly strict or abusive supervision can be included in the definitions of abusive behavior. We cite Tepper (2000, p. 178), who, concerning the gap in expectations at work, defined abusive supervision as a subjective evaluation resting on “subordinates’ perceptions of the extent to which supervisors engage in the sustained display of hostile verbal and nonverbal behaviors, excluding physical contact” (our emphasis). The stress is on subordinates’ perceptions of their supervisors’ conduct toward them and not on their managers’ behavior, per se.

This distinction is particularly germane because the same behavior can be interpreted differently by different workers or even by the same worker at various times (Fischer et al., 2021). This phenomenon, consequently, might influence statistical research on the incidence of behavior abuse. For example, Fischer and colleagues found that in the U.S., 13% of interviewees reported they had experienced psychological abuse at least once a week. However, a review of specific studies on abusive supervision found that less than 2% of these incidents had been officially reported.

Fischer et al. (2021) suggested that the empirical results of their survey were due to statistical analysis challenges. However, we might variously interpret their finding of rarely reported incidences of abusive behavior in the workplace. We propose that the result stems from the fears faced by both the perpetrators and their targets and their reticence to report (as indicated above), and the reservations faced by the relevant organizations facing negative publicity.

#### 2.1.2. Antecedents

We have indicated that abusive behavior arises through possible deficiencies of character and upbringing. Indeed, Cowan (2013) recorded that bullying, in particular, has been perceived by H.R. professionals as a consequence of (a) internal factors, such as management style, personality, and communication skills, and (b) external factors, including (work) culture and norms pervasive in contemporary society (topics that we discuss further emerging from our specific investigation of workplace abuse, see below).

Furthermore, these influential forces were perceived as not under the control of either the bully or the target in unfavorable exchanges. For an instance of a relevant external force, we cite Baker’s (2013) observation that today’s competitive work milieu, replete with promotion schemes and comparative performance review systems, creates a “winner-takes-all” culture that promotes workplace bullying.

#### 2.1.3. Outcomes

As noted, research has documented the outcomes of abusive behavior in the workplace regarding psychological damage to targets and the broader economic implications for the victims of abusive behavior, the workplace, organizational productivity, and the entire economy. For example, Heilbrunn and Itzkovitz (2017) found that economic implications arise for organizations when abused victims embrace extended breaks, slow work, missing workdays, and reporting more workplace accidents – actions that harm productivity.

In that context, and more germane to our discussion, Lazarus and Folkman’s (1984) theory, for instance, throws light on the psychological effect of workplace abuse on (a) horizontal solidarity and (b) employment security. The theory distinguishes between (a) emotional reactions to stressors that inhibit facing up to issues and (b) problem-centered responses that confront specific problems. Emotional reactions reflect a pessimistic outlook on the likelihood of coping with the issue, while the latter response indicates a positive approach to overcoming challenges. Notably, Lazarus and Folkman (1984) distinguished between whether the Source of the abuse stemmed from supervisors or co-workers and their respective weightings on workplace insecurity and horizontal solidarity.

Following this line of discussion, we propose that workplace abuse causes employment insecurity when victims feel they cannot cope with the threats posed by their perceived ill-treatment. Moreover, if the abuser is the worker’s manager, the latter’s behavior is construed as employment insecurity because managers control most of the employee’s socioeconomic resources. On the other hand, abuse by co-workers who do not control resources would not be so construed.

In a related study, Cortina et al. (2001) used the Workplace Incivility Scale (WIS) to measure “experiences of disrespectful, rude, or condescending behaviors from superiors or co-workers” (p. 68) on a seven-item list of incivilities using a four-point scale ranging from 1 = “Almost never” to 7 = “Most of the time.”

The results indicated that uncivil supervision creates a sense of employment insecurity, while incivility by horizontal co-workers does not, although it has some effects. Furthermore, uncivil supervision does not impair or damage the horizontal solidarity between work colleagues; however, if the incivility continues for extended periods, it affects the welfare and social fabric of the workplace (Heilbrunn and Itzkovitz, 2017).

The supervisor, it appears, is the key player to be watched in instances of abusive behavior.

#### 2.1.4. The problem statement

A positive and healthy atmosphere in the workplace is necessary for the well-being of employees while the impact of a workplace disruption on working patterns has a negative influence on all working actors. The present study aims to address this issue.

The statistics (Caillier, 2021) presented in the introduction section provide evidence of the negative influence of workplace bullying (WPB) on the workplace.

The general problem we are addressing is workplace bullying (WPB).

Specifically, we are trying to identify managerial abuse in organizations regarding the type of organization (public/private), gender of the perpetrator, gender of the target, and the managerial role, of each of the abuse items and by that to suggest directions to facilitate the diminution of behavioral abuse in the workplace.

Though abusive behavior in organizations appears to be a widespread phenomenon, previous publications have not addressed the full range of abusive behaviors but rather solely bullying, and have not looked into particular difference factors. Therefore, the gap this study attempts to fill is by referring to the full range of abusive behaviors rather than solely bullying, as well as examining particular difference factors that weren’t examined in previous studies.

### 2.2. Our investigation

Finally, in an additional attempt to conclude supervisors’ abusive behavior in its various manifestations, we conducted an exploratory study in Israel, employing Tepper’s Abusive Supervision Survey Questionnaire [ASSQ]. This exploratory investigation is timely because, despite Israel’s stringent regulations, workplace harassment has not lessened in recent years, and no law is yet in place that frames bullying or harassment as an actionable cause for a claim and compensation.

We focused on the supervisor-subordinate dimension because conclusions drawn from the aforementioned studies and a more recent investigation (Salton Meyer and Ein-Dor, 2021) confirm that the incidence and degree of deleterious effects of supervisor-employee abusive behavior are of more significance than that of colleague-colleague abuse.

Notably, ASSQ is a valuable tool with which (1) to track incidences of abusive behavior in organizations and (2) to provide data that contribute toward mechanisms that attenuate the trust gap at work. Furthermore, we propose that ASSQ taps into the three components of abusive behavior described above: incivility, harassment, and bullying.

In passing, we note that Tepper’s questionnaire may also be used regarding employer/employee behavior toward contingent workers not on the payroll, such as independent contractors, freelancers, other outsourced workers, and even job seekers and job applicants.

In this investigation, we additionally chose to focus on the various incidences of abusive behavior managers display toward their subordinates in organizations, with specific attention to (1) the managerial/supervisory role, (2) the victims’ gender, (3) managers’ gender, and (4) type of organization (public vs. private),

#### 2.2.1. Procedure

Employees of two large organizations (the Katzrin Local Council and Tigbur Ltd.) and another small national organization in Israel were approached via their WhatsApp groups, requesting their assistance in this investigation. We introduced the questionnaire (translated into Hebrew), noting its anonymity and academic context and that subject participation was voluntary.

#### 2.2.2. Participants

One hundred and five respondents comprised the final sample: men (39%) and women (61%) aged 23–66. 66.7% of the respondents were employed in a public organization, 28.5% in a private organization, and 4.8% in a national organization. Years of education: less than 12 (14.3%); 12 full years (17.1%); tertiary education (10.5%); bachelor’s degree (40%); and master’s degree and above (18.1%). While 28.6% held managerial positions, 71.4% did not. Job tenure ranged between 0.5 and 42 years. The pools of participants in national and private organizations were combined to avoid unbalanced groups. Notably, the gender balance of the managers was men (50.5%) and women (49.5%).

#### 2.2.3. The questionnaire

The ASSQ questionnaire consisted of fifteen statements on a five-point response scale (alpha = 0.97, *M* = 1.81, SD = 1.03):

1. I cannot remember him/her ever using this behavior with me;
2. He/she very seldom uses this behavior with me;
3. He/she occasionally uses this behavior with me;
4. He/she uses this behavior moderately often with me; to
5. He/she uses this behavior very often with me.

#### 2.2.4. Statistical analysis

We conducted a factor analysis to ensure statistical validity. We found, however, that the items did not split into well-formed and distinct factors, so the findings are based on a global variable, “abuse” (KMO = 0.92, *R*^2^ = 69.18). It should be noted that the loadings of the items on one factor were very high (> 0.65), which overcomes the relatively small sample size (MacCallum et al., 1999).

We performed Pearson correlations to examine the relationships between the general latent variable and the abuse items and their intercorrelations (see Table 1). Additionally, we conducted t-tests for independent samples (with Bonferroni corrections for multiple comparisons: see, for example, Vickerstaff et al., 2019) to compare (1) the managerial role with each of the abuse items, (2) respondents’ gender, (3) managers’ gender, and (4) type of organization (public/private), including the general abuse variable (see Tables 2, 3).

**Table 1:** Zero-order Pearson correlation matrix.

	1	2	3	4	5	6	7	8	9	10	11	12	13	14	15
Item 1	(−)														
Item 2	0.93	(−)													
Item 3	0.79	0.82	(−)												
Item 4	0.70	0.79	0.81	(−)											
Item 5	0.63	0.69	0.74	0.84	(−)										
Item 6	0.60	0.65	0.66	0.71	0.68	(−)									
Item 7	0.52	0.59	0.68	0.75	0.66	0.71	(−)								
Item 8	0.43	0.50	0.65	0.78	0.70	0.70	0.84	(−)							
Item 9	0.45	0.56	0.69	0.77	0.71	0.66	0.79	0.87	(−)						
Item 10	0.44	0.55	0.64	0.79	0.68	0.68	0.83	0.85	0.82	(−)					
Item 11	0.38	0.50	0.57	0.74	0.71	0.69	0.74	0.83	0.82	0.87	(−)				
Item 12	0.44	0.50	0.62	0.77	0.71	0.66	0.79	0.83	0.78	0.89	0.90	(−)			
Item 13	0.50	0.60	0.63	0.58	0.56	0.40	0.47	0.48	0.52	0.48	0.44	0.47	(−)		
Item 14	0.46	0.54	0.58	0.61	0.56	0.43	0.52	0.59	0.60	0.56	0.59	0.57	0.58	(−)	
Item 15	0.61	0.66	0.75	0.79	0.64	0.59	0.78	0.76	0.71	0.78	0.70	0.80	0.62	0.65	(−)
**Abuse (latent)**	0.69	0.77	0.84	0.92	0.84	0.80	0.87	0.89	0.88	0.89	0.86	0.88	0.65	0.70	0.87

All correlations are significant at *p* < 0.001; a full description of items may be found in Tables 2, 3.

**Table 2:** Results for comparing gender of managers and employees based on the items.

	Employees			*t*-test	Managers			*t*-test
	Men (*n* = 53)		Women (*n* = 52)		Men (*n* = 41)		Women (*n* = 64)	
1. Ridicules me	1.73 (1.28)	>	1.31 (0.73)	2.13^*^	1.74 (1.18)	=	1.33 (0.83)	1.72
2. Tells me my thoughts or feelings are stupid	1.80 (1.29)	>	1.36 (0.80)	2.18^*^	1.96 (1.24)	>	1.38 (0.84)	2.05^*^
3. Gives me the silent treatment	1.98 (1.33)	>	1.48 (0.87)	2.29^*^	2.02 (1.28)	>	1.25 (0.86)	2.79^***^
4. Puts me down in front of others	1.95 (1.34)	>	1.44 (0.97)	2.27^*^	1.91 (1.21)	>	1.38 (0.91)	3.61^**^
5. Invades my privacy	1.93 (1.21)	>	1.47 (0.99)	2.12^*^	2.36 (1.51)	>	1.62 (1.29)	2.49^*^
6. Reminds me of my past mistakes and failures	2.54 (1.73)	>	1.64 (1.10)	3.24^**^	2.72 (1.68)	>	1.54 (1.04)	2.72^***^
7. Doesn’t give me credit for jobs requiring a lot of effort	2.56 (1.70)	>	1.86 (1.32)	2.37^*^	2.68 (1.63)	>	1.56 (1.11)	4.31^***^
8. Blames me to save himself/herself embarrassment	2.49 (1.66)	>	1.89 (1.35)	2.02^*^	2.55 (1.44)	>	1.56 (0.98)	4.12^***^
9. Breaks promises he/she makes	2.39 (1.50)	>	1.84 (1.16)	2.10^*^	2.57 (1.42)	>	1.33 (0.86)	4.12^***^
10. Expresses anger at me when he/she is mad for another reason	2.29 (1.52)	>	1.73 (1.14)	2.14^*^	2.62 (1.47)	>	1.38 (0.87)	5.40^***^
11. Makes negative comments about me to others	2.51 (1.57)	>	1.69 (1.10)	3.17^**^	2.60 (1.51)	>	1.35 (0.88)	5.24^***^
12. Is rude to me	2.41 (1.56)	>	1.70 (1.19)	2.64^**^	1.66 (1.09)	>	1.38 (0.99)	5.19^***^
13. Does not allow me to interact with my coworkers	1.61 (1.16)	=	1.47 (0.98)	0.67	1.87 (1.06)	=	1.25 (0.65)	1.35
14. Tells me I’m incompetent	1.66 (1.06)	=	1.50 (0.84)	0.85	2.40 (1.25)	>	1.33 (0.88)	3.60^***^
15. Lies to me	2.07 (1.23)	=	1.73 (1.17)	1.42	2.22 (1.08)	>	1.40 (0.78)	5.07^***^
**Abuse (latent)**	2.13 (1.17)	>	1.61 (0.88)	2.60^*^	1.74 (1.18)	>	1.33 (0.83)	4.47^***^

*p < 0.05, **p < 0.01, ***p < 0.001.

**Table 3:** Results for comparing types of organizations and managerial positions based on the items.

	Organization			*t*-test	Position			*t*-test
	Private (*n* = 35)		Public (*n* = 70)		Non-managerial (*n* = 41)		Managerial (*n* = 64)	
1. Ridicules me	1.80 (1.05)	>	1.31 (0.94)	2.40^*^	1.48 (0.96)	=	1.47 (1.11)	0.06
2. Tells me my thoughts or feelings are stupid	2.03 (1.12)	>	1.29 (0.90)	3.66^***^	1.53 (0.99)	=	1.53 (1.17)	0.00
3. Gives me the silent treatment	2.31 (1.13)	>	1.36 (0.93)	4.61^***^	1.67 (1.07)	=	1.70 (1.18)	0.14
4. Puts me down in front of others	2.43 (1.33)	>	1.24 (0.81)	5.66^***^	1.72 (1.26)	=	1.43 (0.82)	1.15
5. Invades my privacy	2.23 (1.33)	>	1.36 (0.83)	4.11^***^	1.72 (1.17)	=	1.47 (0.90)	1.07
6. Reminds me of my past mistakes and failures	2.83 (1.52)	>	1.57 (1.21)	4.59^***^	2.15 (1.54)	=	1.60 (1.10)	1.77
7. Doesn’t give me credit for jobs requiring a lot of effort	3.17 (1.52)	>	1.61 (1.22)	5.67^***^	2.11 (1.48)	=	2.20 (1.63)	0.28
8. Blames me to save himself/herself embarrassment	3.37 (1.59)	>	1.50 (0.97)	7.45^***^	2.24 (1.59)	>	1.83 (1.21)	1.26^*^
9. Breaks promises he/she makes	3.14 (1.42)	>	1.51 (0.86)	7.30^***^	2.12 (1.37)	=	1.90 (1.21)	0.77
10. Expresses anger at me when he/she is mad for another reason	3.00 (1.37)	>	1.43 (0.94)	6.89^***^	2.01 (1.40)	=	1.80 (1.13)	0.74
11. Makes negative comments about me to others	3.20 (1.39)	>	1.41 (0.86)	8.11^***^	2.20 (1.44)	>	1.53 (0.97)	2.33^*^
12. Is rude to me	3.11 (1.45)	>	1.41 (0.94)	7.24^***^	2.12 (1.48)	=	1.63 (1.07)	1.64
13. Does not allow me to interact with my coworkers	1.91 (1.12)	>	1.33 (0.96)	2.79^***^	1.44 (0.93)	=	1.73 (1.28)	1.30
14. Tells me I’m incompetent	2.29 (1.02)	>	1.20 (0.63)	6.74^***^	1.57 (0.90)	=	1.53 (1.01)	0.20
15. Lies to me	2.74 (1.04)	>	1.43 (1.03)	6.15^***^	1.88 (1.16)	=	1.83 (1.32)	0.18
**Abuse (latent)**	2.64 (0.97)	>	1.40 (0.78)	7.09^***^	1.86 (1.07)	=	1.68 (0.91)	0.83

*p < 0.05, **p < 0.01, ***p < 0.001.

#### 2.2.5. Results

Table 1 reveals that all the correlations are high, positive, and significant. Below is a list of items that predicted abuse in descending order. The numbers 1–15 in square brackets denote the item numbers as shown in Tables 2, 3:

- [4] Puts me down in front of others (*r* = 0.92).
- [8] Blames me to save himself/herself embarrassment & [10] expresses anger at me when he/she is mad for another reason (*r* = 0.89).
- [9] Breaks promises he/she makes, and [12] is rude to me (*r* = 0.88).
- [7] Doesn’t give me credit for jobs requiring a lot of effort, and [15] lies to me (*r* = 0.87).
- [11] Makes negative comments about me to others (*r* = 0.86).
- [3] Gives me the silent treatment and [5] invades my privacy (*r* = 0.84).
- [6] Reminds me of my past mistakes and failures (*r* = 0.80).
- [2] Tells me my thoughts or feelings are stupid (*r* = 0.77).
- [14] Tells me I’m incompetent (*r* = 0.70).
- [1] Ridicules me (*r* = 0.69).
- [13] Does not allow me to interact with my co-workers (*r* = 0.69).

#### 2.2.6. Discussion of findings

Because the mean of the reported abuse was relatively low (1.81), we conclude that the incidence of abusive behavior in the respondents’ organizations is not high. We explain the result by presuming that a particular behavior interpreted as abuse by one individual is not deemed so by another. Furthermore, circumstances might also prevail: a statement perceived as incidental in one situation might be considered harassment in another. However, the meager reporting does not necessarily mean that abusive behavior is not extant. We reiterate that the finding matches Fischer et al.’s (2021) conclusion that abusive supervision is underreported. As noted, these researchers saw that phenomenon as a statistical-research challenge rather than an indication that the phenomenon is nonexistent; we variously implied that the fears associated with the exposure of the abuse mitigated against reporting.

Specific to the four areas of association we investigated, our findings indicate that workers reported more cases of abuse than managers, but, again, the mean differences between managers and employees were minor (a difference of between 0 and 0.67 in the mean scores, in absolute value). On the other hand, men reported more cases of abusive behavior than women. Again, however, the differences found were insignificant and cannot serve to form conclusions that indicate a trend.

Women managers exhibited fewer abusive supervisory behaviors than men. However, the mean differences between men and women were modest (between 0.28 and 1.25 difference in the mean scores, in absolute value). Despite finding no backing for this outcome in the literature, the finding appears to be explained by Miller et al. (2012) supposition that in the organizational arena, the female management style is characterized by a normative ethical theory, “ethics of care” Gilligan (1982) – an approach that centers on interpersonal relations and cares as a virtue. Consequently, women are more likely than men to be creative, supportive, and innovative when supervising subordinates, even involving their workers in decision-making.

The lower incidence of reported abuse among female managers could also be understood in line with studies that indicate that men display and report aggression more frequently than women (e.g., Tavris and Wade, 1984; kogut and Zander, 1992; Hershcovis and Barling, 2007), that women reflect more stable personality dispositions, and that men are more likely than women to aggress against others without provocation (Björkqvist et al., 1994) – seemingly playing out their expected respective stereotypical roles in society (Kawakami et al., 1999; Fiske et al., 2002). However, because the differences found in all the variables in our investigation investigated were not significant, we cannot conclude that the results indicate a trend.

We further found that abusive behavior was less prevalent in the two public concerns than in the private organization. Again, we found no support for this result in the literature, but we assume that regulation in public organizations accounts for reducing the possibilities of abuse.

## 3. Conclusions and recommendations

There is increasing awareness that abusive behavior in the workplace is a widespread social phenomenon (Vault Platform, 2021). Given the changing and developing labor market, the importance of addressing this topic is apparent (Dumitru, 2016). In Israel, for instance, there is as yet no appropriate solution for this issue, which has organizational, economic, and legal consequences beyond the psychological and mental health implications.

We have recorded, however, that reports of the phenomenon are meager, despite increasing awareness, and thus we presumed that various phenomena account for that finding. They include: (1) the same behavior could be interpreted as abuse by one individual but not by another; (2) Statements perceived as incidental in one circumstance are regarded as harassment in others; and (3) There is a propensity not to disclose instances of abuse due to the fear of consequences.

### 3.1. Implications for future research and recommendations

On the research level, we propose developing methodological tools adapted to further exploration of abusive behavior. As noted by a reviewer of this paper, we need to look more deeply into the sources (antecedents) of abusive behavior at work. Thus, future research endeavors could consider the variable of cultural values on the relationship between abusive supervision and the workplace and their impact on employees, customers, shareholders, and the community. We could envisage, for example, the effect of racial or sexual prejudice on the incidence of abuse at work.

Furthermore, based on the findings in this paper, future investigations could shed further light on whether female managers display more or less abusive behavior at work than male managers. For instance, despite the overarching conclusion that women are less aggressive than men, when operative in an all-male milieu, the answer could be “more abusive” (Miller et al. 2012; News, 2018). Could the reason be the existence of the “Queen bee syndrome” whereby women with authority in a male-dominated environment treat subordinates more critically: that rigid stance then flies in the face of the “ethics of care” theory discussed above.

Researchers could also further investigate how various HRM practices, such as organizational hierarchies, performance management procedures, and tournament promotion, foster abusive behavior at work.

Additionally, research into the impact of the narrower organizational culture on behavioral abuse (e.g., staff cohesion, LMX) is imperative in efforts to attenuate abusive workplace behavior. We argue that despite the lesser reported manifestation of abuse by co-workers accounted for in the cited research findings, abusive behavior between employees (i.e., antecedents, manifestations, process) deserves to be further investigated – if for no other reason, practical recommendations to circumvent abuse can be derived from the research findings.

On a local level, management can institute a series of deterrence mechanisms that include tools to prevent inappropriate behaviors, such as punishment of abusers, correlated to the degree of exposure to the abuse by the victim. But, unless the root causes of workplace abuse are investigated, sanctions and legal remedies will never be sufficient.

As stated in the Introduction, we recommend cultivating a positive organizational culture that eradicates the “trust gap” and encourages employees to speak up without fear of retribution or threat to their jobs. We urge companies – so much more aware of workplace sexual abuse and the need for diversity and sustainability in their organizations to raise consciousness and provide practical tools for eliminating work abuse and promoting staff cohesion. Examples include training measures, workshops, and sensitive supervision.

We add that on the community and legal fronts, public discourse should continue to raise awareness of the issue and the desired outcome – increasing workers’ protection and protecting their dignity – while governments should promote further relevant legislation to protect workers from abuse.

To this end, we further recommend the appointment of an officer in charge of eradicating abuse in the organization, to whom employees can complain and who, additionally, would help courts identify abusive behavior when employees file lawsuits against a perpetrator. We also propose assigning court-appointed experts to examine abuse allegations utilizing the statements in Tepper’s (2000) questionnaire. The experts would assist the courts (and the organizations) in establishing the incidence of abusive activity, as well as its substantive nature (validity) and frequency in the workplace, especially where particular behavioral aspects of abuse are not (yet) included in legal definitions.

Ultimately, all these measures aim to protect workers’ dignity, the integrity of the organizations, and the welfare of society as a whole.

## Data availability statement

The raw data supporting the conclusions of this article will be made available by the authors, without undue reservation.

## Ethics statement

The ethics committee of Peres Academic Center headed by Professor Amos Drory has approved the study following a diligent examination of all ethical considerations. The approval carries the ordinal number of 1001 and is dated 27th of December 2021: Abusive Workplace Behavi. The patients/participants provided their written informed consent to participate in this study.

## Author contributions

All authors listed have made a substantial, direct, and intellectual contribution to the work and approved it for publication.

## Conflict of interest

The authors declare that the research was conducted in the absence of any commercial or financial relationships that could be construed as a potential conflict of interest.

The handling editor EF declared a past co-authorship with the author AT.

## Publisher’s note

All claims expressed in this article are solely those of the authors and do not necessarily represent those of their affiliated organizations, or those of the publisher, the editors and the reviewers. Any product that may be evaluated in this article, or claim that may be made by its manufacturer, is not guaranteed or endorsed by the publisher.
